# Episodic Src activation in uveal melanoma revealed by kinase activity profiling

**DOI:** 10.1038/sj.bjc.6605172

**Published:** 2009-06-30

**Authors:** W Maat, M el Filali, A Dirks- Mulder, G P M Luyten, N A Gruis, L Desjardins, P Boender, M J Jager, P A van der Velden

**Affiliations:** 1 Department of Ophthalmology, Leiden University Medical Center, PO Box 9600, 2300 RC Leiden, The Netherlands; 2 Hogeschool Leiden, Zernikedreef 11, 2333, CK Leiden, The Netherlands; 3 Department of Dermatology, Leiden University Medical Center, PO Box 9600, 2300 RC Leiden, The Netherlands; 4 Department of Ophthalmology, Institute Curie, 26 rue d′Ulm 75248 Paris, France; 5 PamGene, Nieuwstraat 30, 5211 NL Den Bosch, The Netherlands

**Keywords:** uveal melanoma, tyrosine kinase, ERK, Src

## Abstract

**Background:**

The RAS/RAF/MEK/ERK pathway is involved in the balance between melanocyte proliferation and differentiation. The same pathway is constitutively activated in cutaneous and uveal melanoma (UM) and related to tumour growth and survival. Whereas mutant BRAF and NRAS are responsible for the activation of the RAS/RAF/MEK/ERK pathway in most cutaneous melanoma, mutations in these genes are usually absent in UM.

**Methods:**

We set out to explore the RAS/RAF/MEK/ERK pathway and used mitogen-activated protein kinase profiling and tyrosine kinase arrays.

**Results:**

We identified Src as a kinase that is associated with ERK1/2 activation in UM. However, low Src levels and reduced ERK1/2 activation in metastatic cell lines suggest that proliferation in metastases can become independent of Src and RAS/RAF/MEK/ERK signalling. Inhibition of Src led to the growth reduction of primary UM cultures and cell lines, whereas metastatic cell line growth was only slightly reduced.

**Conclusion:**

We identified Src as an important kinase and a potential target for treatment in primary UM. Metastasis cell lines seemed largely resistant to Src inhibition and indicate that in metastases treatment, a different approach may be required.

Uveal melanoma (UM) is a rare neoplasm that arises from melanocytes in the eyes. It usually affects people in their sixties with an incidence rate of ∼6–8 new cases per million per year among Caucasians (Egan *et al*, 1988; Singh and Topham, 2003). Little is known about the molecular pathogenesis of UM compared with cutaneous melanoma (CM). Cutaneous melanoma and UM share the same embryonic origin and similar histological features, but mutations that regulate proliferation and cause a loss of cell-cycle control in CM can hardly be found in UM. Whereas p16-regulated cell cycle control is targeted by the deletion of chromosome 9p or by the mutation of *CDKN2A* in CM, most of the UM cell lines posses a wild-type p16-encoding gene that is, however, not expressed because of the epigenetic modification of the *CDKN2A* gene (van der Velden *et al*, 2001). The same may be true for the activation of the RAS-RAF-MEK-ERK or the classical mitogen-activated protein kinase (MAPK) pathway. The MAPK activation is crucial for the development of melanocytic neoplasia, and a constitutive activation of this pathway has been associated with many different types of cancer (Goding, 2000; Reddy *et al*, 2003). In CM, the activation of the MAPK pathway has been shown to occur by a variety of mechanisms, including autocrine growth factor stimulation and mutation of the *NRAS* (20% of cases) and *BRAF* (60% of cases) genes (van Elsas *et al*, 1995; Davies *et al*, 2002; Satyamoorthy *et al*, 2003). The *BRAF* mutations have only rarely been reported in UM and activating mutations in *NRAS*, which are found in 25% of all cancers, have never been reported (Mooy *et al*, 1991; Soparker *et al*, 1993; Cohen *et al*, 2003; Cruz III *et al*, 2003; Edmunds *et al*, 2003; Rimoldi *et al*, 2003). However, we and others have found that UM is heterogeneous and that, with more sensitive techniques, the percentage of mutant *BRAF*-positive UM may be higher (Janssen *et al*, 2008; Maat *et al*, 2008). The lack of mutations in the majority of cells is in contrast with immunohistochemistry and western blot analysis, which have shown activation of ERK1/2 in most UM (Rimoldi *et al*, 2003; Weber *et al*, 2003; Zuidervaart *et al*, 2005). Nevertheless, the pharmacological inhibition of MAPK/ERK kinases 1 and 2 (MEK1/2) and the genetic targeting of BRAF with siRNA resulted in a reduced proliferation of UM cell lines (Lefevre *et al*, 2004; Calipel *et al*, 2006). This indicates that although mutations are absent, the RAS/RAF/MEK/ERK pathway is essential for UM growth and suggests that an upstream factor is involved in autonomous UM proliferation. Recently, c-Kit was shown to be upregulated in UM and involved in an autocrine loop that also involved the RAS/RAF/MEK/ERK pathway (Lefevre *et al*, 2004). An incomplete response to c-Kit inhibition indicates that additional factors are involved (Hofmann *et al*, 2009). In addition, the *GNAQ* gene was shown to be mutated in almost half of UM (Van Raamsdonk *et al*, 2008). GNAQ is part of the G-protein heterotrimer and represents the GTP-binding part that couples GPCR signalling to MAPK activation, which marks it as a potential therapeutic target. However, targeting downstream signalling molecules may be just as effective as they may be shared with other mutant pathways. Tyrosine kinase activity profiling in UM was used to explore the involved kinases. On the basis of a UM cell line and two related metastasis cell lines, which revealed reduced ERK1/2 activation in metastases, we were able to identify Src as a crucial upstream tyrosine kinase for ERK1/2 activation in primary UM. Unfortunately, metastasis cell lines seemed less dependent on Src and may indicate that metastasis may require an alternative approach for intervention.

## Materials and methods

### Cell lines and tumour material

A total of 11 cell lines derived from primary UM (92.1; OCM-1, -3 and -8; Mel-202, -270, -285, -290) and UM metastases (OMM-1, -2.3 and -2.5) were analysed for kinase activity (Kan-Mitchell *et al*, 1989; Waard-Siebinga *et al*, 1995; Luyten *et al*, 1996; Chen *et al*, 1997). UM cell lines were cultured in RPMI 1640 medium (Gibco, Paisley, Scotland) supplemented with 3 mM L-glutamine (Gibco), 2% penicillin/streptomycin and 10% FBS (Hyclone, Logan, UT, USA). Primary UM was cultured in Amniochrome Pro Medium (Lonza Group Ltd, Basel, Switzerland). All cell cultures were incubated at 37°C in a humidified 5% CO_2_ atmosphere.

Cell lysates were obtained by lysing cells in M-PER Mammalian Protein Extract Reagent (Pierce, Rockford, IL, USA), supplemented with 1% Halt Protease Inhibitor Cocktail, EDTA-free (Pierce) and 1% Halt Phosphatase Inhibitor Cocktail (Pierce). Protein concentrations were measured by using the BCA Protein Assay kit (Pierce). Cell lysates were also acquired from three fresh primary UM samples obtained by enucleation and from three liver metastases of three different patients, in whom the diagnosis was confirmed.

### Phospho-MAPK array

The Human Phospho-MAPK Array (R&D Systems, Abingdon, UK) was used to simultaneously detect the relative levels of nine MAP kinases and nine other serine/threonine kinases in cell lines, in primary UM and in liver metastasis. In this array, capture and control antibodies were spotted in duplicate on nitrocellulose membranes. Experiments were carried out according to the manufacturer's guidelines. In short, cell lysates were diluted and incubated with the array. After binding of both phosphorylated and unphosphorylated kinases, unbound material was washed away. A cocktail of phospho-site-specific biotinylated antibodies was used to detect phosphorylated proteins through streptavidin-horseradish peroxidase and chemiluminescence. X-ray films of the blots were scanned and analysed using G-boxHR (Syngene, Frederick, MD, USA). Control spots with mouse, goat and rabbit antibodies were used for background correction.

### PamGene tyrosine kinase array

Experiments were carried out using a 4-array semi-automated system (PamStation 4, PamGene, 's-Hertogenbosch, The Netherlands) designed for processing PamChip-4 arrays. The PamChip Tyrosine Kinase Array (PamGene) contains 144 phospho-peptides, immobilised on a porous microarray surface through the peptide N terminus, representing tyrosine kinase substrates. Each array was blocked with 0.2% bovine serum albumin (BSA), fraction V (Calbiochem Immunochemicals, Merck KGaA, Darmstadt, Germany) by pumping it through the porous microarray for 30 cycles of 30 s. Thereafter, each array was washed thrice for 8 s with 1 × ABL Protein Tyrosine Kinase Reaction Buffer solution (New England Biolabs, Ipswich, MA, USA). Next, incubation was carried out at 30°C with the reaction mix, containing 5 *μ*g cell lysate, 4 *μ*l 100 × BSA (New England Biolabs), 0.4 *μ*l 10 mM ATP (Sigma-Aldrich, Zwijndrecht, The Netherlands) and 0.5 *μ*l 1 mg ml^−1^ monoclonal anti-phosphotyrosine FITC conjugate (clone Py20, Exalpha Biologicals, Maynard, MA, USA), adjusted to 40 *μ*l with distilled H_2_O. The sample was pulsed back and forth through the porous material for 45 cycles, which is coupled to the base of a well to maximise reaction kinetics and to reduce analysis time. At every fifth pump cycle, a 16-bit TIFF image was taken with a built-in CCD camera (PamGene).

Blocking experiments were carried out with Src family-selective tyrosine kinase inhibitors, PP1, PP2 (Biomol International, LP, of Plymouth Meeting, PA, USA) and PP3 (the inactive analogue, Calbiochem), at an end concentration of 10 *μ*M in line with a large body of literature. Each particular inhibitor was mixed with lysates of cell lines and tissue, together with the reaction mix just before incubation on the array.

Acquired data from the PamStation 4 were captured with the supplied software package BioNavigator (Version 0.3.1; PamGene). For the purpose of finding differentially phosphorylated substrates, data were imported in the LIMMA package (Bioconductor.org) and we applied the empirical Bayes method (Smyth, 2004). Background subtracted data were normalised for differences between experiments, and substrates and *P*-values of 0.05 or less were corrected for multiple testing using the Benjamini and Hochberg correction. Substrates with a corrected *P*-value of 0.05 or less were assumed to be significant.

### Western blot analysis

Cell lysates (10 *μ*g) were separated on 12.5% SDS–PAGE gels, and proteins were transferred to Hybond-polyvinyldifluoride membranes (Amersham Biosciences, Buckinghamshire, UK). The membranes were blocked with 5% skim milk in a PBS-Tween 0.1% solution and probed at room temperature for 1 h with antibodies specific to each antigen: phospho-Src (Tyr527; dilution 1 : 1000), phospho-Src family (Tyr416; dilution 1 : 1000) and Src (36D10; dilution 1 : 1000) antibody (all from Cell Signaling Technology, Hertfordshire, UK). An antibody against actin (Abcam, Cambridge, UK) was used as a loading control. Membranes were subsequently incubated at room temperature with horseradish peroxidase-conjugated IgG anti-mouse or anti-rabbit secondary antibodies for 1 h. Supersignal West Femto ECL (Pierce) was used to visualise protein bands on the membrane.

### siRNA treatment

Sub-confluent cell cultures were grown without antibiotics 24 h before transfection in RPMI 1640 medium. A mixture of Lipofectamine 2000 (Invitrogen, Carlsbad, CA, USA) and two different siRNA constructs (40 nM) were incubated in the standard medium with reduced serum (1%), according to the manufacturer's instructions. The siRNA constructs (Stealth) were predesigned and validated (∼70% knockdown) by the manufacturer (Invitrogen). After 24 and 48 h, the cells were harvested and RNA and protein lysates were prepared.

### WST-1 assay

Cell proliferation in response to PP1 (10 and 50 *μ*M) was measured by mitochondrial function using the WST-1 proliferation reagent (Sigma-Aldrich) as described earlier (Narayanan *et al*, 2005). This assay measures tetrazolium reductase activity in the mitochondria, which serves as a measure of cell viability. In short, 96-well plates were filled with 1250 UM cells per well. At 1 or 3 days (tumour 1–5) and 1 or 6 days (92.1; OCM-1, -3 and -8; Mel-202, -270, -285, -290, OMM-1, -2.3 and -2.5) after treatment, the WST-1 reagent was added and absorbance was measured at 450 nm on a multiwell spectrophotometer. The median and standard error of eight wells were taken at each time and dosage point.

### Quantitative PCR

The cell lines (92.1; OCM-1, -3 and -8; Mel-202, -270, -285, -290, OMM-1, -2.3 and -2.5) were analysed for Src gene expression. Primers for Src and the reference gene, *β*-actin, were developed with Beacon Designer software (Premier Biosoft, Palo Alto, CA, USA). Primer sequences for Src: 5′-GCTGCGGCTGGAGGTCAAG-3′ (forward) and 5′-AGACATCGTGCCAGGCTTCAG-3′ (reverse). Primer sequence for *β*-actin: 5′-CGGGACCTGACTGACTACCTC-3′ (forward) and 5′-CTCCTTAATGTCACGCACGATTTC-3′ (reverse). The PCR reaction settings were 95°C for 5 min, then 40 cycles at 96°C for 15 s and at 60°C for 45 s. The DNA melting point of the amplicons was acquired by measuring the fluorescence of SYBR Green (Bio-Rad, Hercules, CA, USA) during a linear temperature transition from 70 to 97°C at 0.2°C each for 10 s with accompanying software (Bio-Rad).

## Results

### ERK1/2 activation in UM

**Figure 1:**
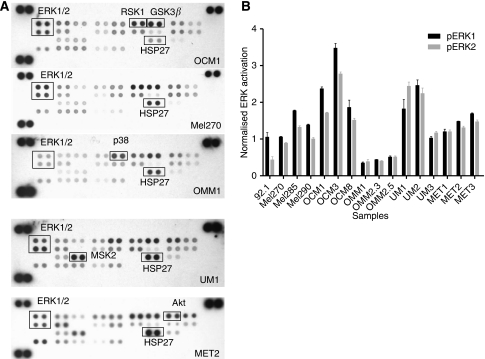
The MAPK activation in primary UM and UM metastases was studied with a MAPK antibody array. We observed uniform HSP27 phosphorylation in both cell lines and tissue samples, except in OCM1, -3 and -8 (**A**). Activated ERK1/2 was normalised with HSP27 and shown to be low in UM metastases (MET1-3), whereas metastatic cell lines just passed the background (OMM1, -2.3, -2.5) (**B**).

An antibody array was applied to investigate the MAPK pathway in 10 UM cell lines, in three primary UM and three UM metastasis. We observed a uniform HSP27 phosphorylation, with the exception of in three UM cell lines (OCM1, -3, -8). UMs displaying activated ERK1/2 as well as phosphorylated HSP27 were most common, whereas signals for phosphorylated ERK1/2 were low in metastasis tissue (MET1-3) and metastatic UM cell lines (OMM1, OMM2.3 and OMM2.5) (Figure 1). Remarkably, two of the metastatic cell lines (OMM2.3, OMM2.5) are derived from the same patient as cell line Mel270 but contained far less activated ERK1/2.

### Differential kinase activity in UM

Reduction of ERK1/2 activation in metastatic cell lines compared with that in primary UM cell lines provides a model to identify the underlying mechanism of ERK1/2 activation in the absence of *BRAF* and *NRAS* mutations.

**Figure 2:**
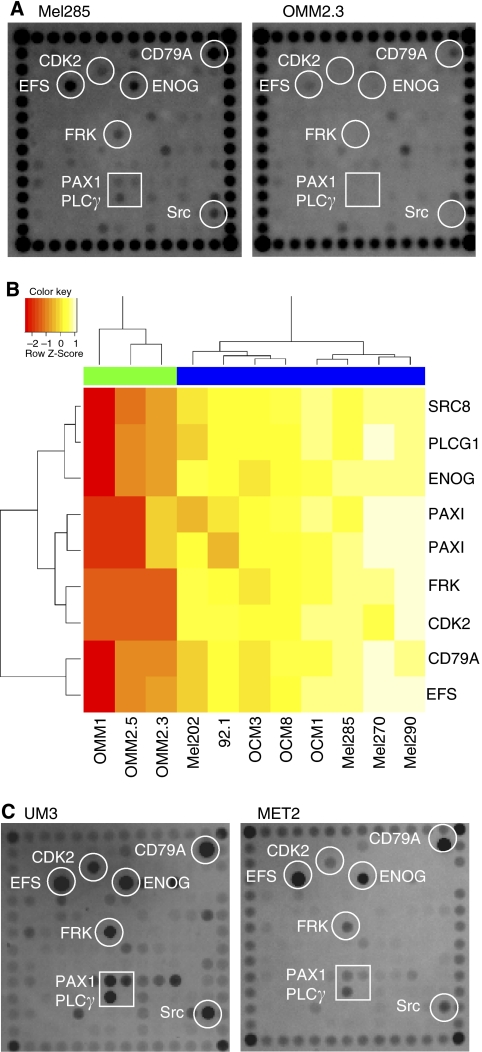
Tyrosine kinase activity was measured with an array of peptide substrates. Two representative examples of a UM cell line and a metastatic cell line (**A**). Analysis with eBayes identified nine substrates, representing eight proteins, to be significantly (*P*=0.01) differentially phosphorylated between UM and metastatic cell lines (**B**). UM (UM1-3) tyrosine kinase activity is high compared with liver metastasis (MET1-3) (two representative arrays are shown) (**C**).

To investigate whether a kinase is differentially activated between primary UM cell lines and metastatic UM cell lines, we used peptide-based tyrosine kinase arrays (Lemeer *et al*, 2007). The UM cell lines displayed a high kinase activity, whereas the metastatic UM cell lines displayed a low kinase activity, although the same amount of lysate was incubated (Figure 2A). After normalisation, we could analyse the kinase data and identify nine substrates that were significantly differentially phosphorylated between primary and metastatic UM cell lines (Figure 2B, Table 1). Primary UM and metastatic tissue also showed differential phosphorylation of these nine peptides, although not as clearly as observed in the cell lines (Figure 2C).

### Candidate kinase: Src

**Figure 3:**
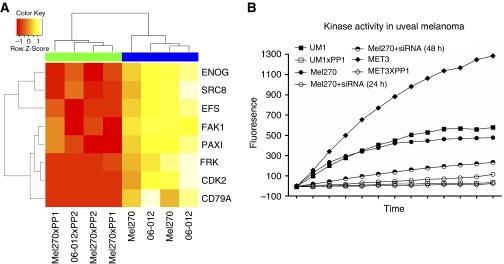
UM1 (06-12) and Mel270 treatment with Src inhibitors (PP1/PP2) identified eight substrates with a significant reduction in phosphorylation (**A**). The inhibition of EFS peptide phosphorylation by genetic (Src siRNA) and pharmacological means (PP1) in cell line Mel270, and PP1 treatment of cell lysates of UM (UM1) and metastasis tissue (MET3) (**B**).

We identified nine peptides derived from eight proteins that were differentially phosphorylated between primary and metastatic cell line lysates. On the basis of a literature search, we identified candidate tyrosine kinases for eight out of nine peptides (Table 1) (Cooper *et al*, 1983; Thomas and Brugge, 1997; Koike *et al*, 2003; Diella *et al*, 2004). Among the candidates, Src and Src family members were most prominent. To validate the candidacy of Src, we performed *in vitro* inhibition experiments with the Src-kinase-specific inhibitors PP1 and the PP1 analogue, PP2. We added PP1 and PP2 (10 *μ*M) to lysates of primary UM tissue and of a primary UM cell line and measured the inhibitory effect of these Src inhibitors using kinase array (Figure 3A). A total of seven out of nine substrates that identified Src in the first screen displayed a significantly reduced phosphorylation when PP1 or PP2 were added to lysates of UM1 and Mel270 (Figure 3A). The PLCG1 peptide and one of the PAX1 (Y31) substrates did not reach significance but were still phosphorylated at a reduced level after PP1 and PP2 treatments. The peptide representing FAK1 Y576/Y577 is a genuine substrate for Src, which was not detected in the UM cell line comparison, but phosphorylation was significantly downregulated by PP1 and PP2 treatments. In the control experiment, in which we added the inactive analogue of PP1 (PP3) to cell lysates, we did not observe a loss of kinase activity (not shown).

The kinase activity of metastasis tissue and UM tissue differed marginally (Figure 2C), and incubation with PP1 (10 *μ*M) resulted in a decimation of kinase activity similar to the inhibition that we observed in UM tissue (Figure 3B). To validate Src activity, Mel270 was transfected with two siRNA constructs that target Src and reduced kinase activity (Figure 3B).

### Regulation of ERK1/2 and growth

**Figure 4:**
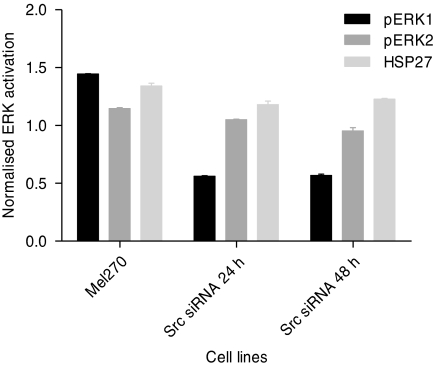
ERK1/2 activation in Mel270 24 and 48 h after transfection with Src siRNA. Phosphorylated HSP27 is included as reference signal.

To investigate whether Src contributes to ERK1/2 activation in Mel270, we analysed the two Src siRNA-transfected cell cultures with the MAPK antibody array. At 24 and 48 h after transfection with Src siRNA, we observed a reduced ERK1 phosphorylation, whereas ERK2 phosphorylation was minimally affected (Figure 4).

**Figure 5:**
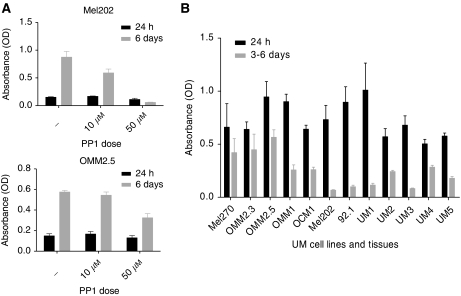
UM cell lines and primary cultures were cultured with PP1 (10 *μ*M and 50 *μ*M). After 24 h and at 3 days (UM cultures) and 6 days (cell lines), viability was tested with the WST-1 assay. Two representative cell culture experiments for which all time points and conditions are shown (**A**). Growth inhibition by PP1 (50 *μ*M) after 24 h and at 3 and 6 days was normalised to the control culture of each individual cell line (**B**).

Whether Src inhibition and consequently a lowered ERK activation in UM cell lines is associated with a reduced growth was investigated with the WST-1 viability assay (Figure 5). All UM cell lines showed a PP1-dose and time (1–6 days)-dependent reduction in cell viability but the magnitude of the response differed widely. In general, the metastatic UM cell lines were less affected by PP1. We also determined the growth inhibition rate of PP1 in cultures of five primary UM cell cultures and observed an increased sensitivity to PP1 treatment compared with the cell lines. We had to take samples at day 3 of PP1 treatment because, thereafter, massive cell death occurred (Figure 5B).

### Src protein is reduced in metastasis cell line

**Figure 6:**
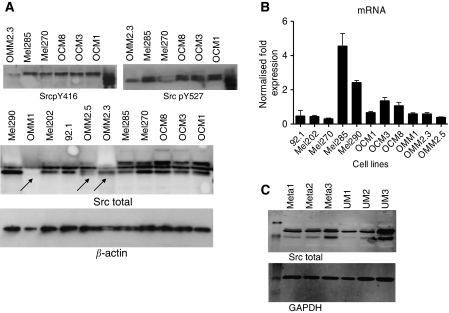
Western analysis of Src in the UM cell lines for activating phosphorylation (Y416), inactivating phosphorylation (Y527) and total Src expression (**A**). Src gene expression measured by qPCR varied widely but did not correlate with a variation in protein expression (**B**). UM and metastasis tissue all displayed a medium Src kinase expression, except for UM3, which presents a high level of expression (**C**).

Src is regulated by the phosphorylation of tyrosine residues at position 416 (Y416) and 527 (Y527). The expression of phosphorylated Src Y416, which is associated with an active conformation, was low in the metastatic UM cell lines (Figure 6A). Surprisingly, the phosphorylation of Y527, which is associated with an inactive conformation, was also low, and a subsequent analysis indicated that Src expression is low in metastatic UM cell lines. Therefore, the difference between kinase activity in metastatic cell lines (OMM1, OMM2.3 and OMM2.5) and UM cell lines (OCM1, OCM3, OCM8, Mel202, Mel270, Mel285, Mel290 and 92.1) seems to be the result of a difference in Src expression.

To investigate the origin of a lowered Src expression, we performed a gene expression analysis (Figure 6B). Src gene expression varied widely in the cell lines and in the metastatic cell lines, but a correlation between protein and gene expression was not observed in UM cell lines.

A western analysis of Src expression in UM and metastasis tissue revealed a very high Src expression in only one out of three primary UM, whereas all three metastasis tissues displayed medium expression of Src protein (Figure 6C).

## Discussion

Constitutive activation of ERK1/2 has often been reported for UM (Rimoldi *et al*, 2003; Weber *et al*, 2003; Zuidervaart *et al*, 2005). Using a more quantitative approach, we distinguished a decrease in active ERK1/2 in metastatic cell lines and in fresh liver metastasis, suggesting a loss of ERK1/2 activation during UM progression. The latter is unexpected as ERK1/2 activation is generally associated with mitogen signalling and is known to determine malignant potential *in vitro*. However, in endometrial and breast cancer, ERK1/2 activation has been associated with a good prognosis (Milde-Langosch *et al*, 2005) (Mizumoto *et al*, 2007). A possible explanation is provided by the observation that ERK1/2 is involved in oncogene and stress-induced senescence (Serrano *et al*, 1997; Stott *et al*, 1998). This mechanism is thought to be an important defence for cells that are at risk of neoplastic transformation and need to be circumvented by tumour cells in order to proliferate. Loss of activated ERK1/2 may not only relieve the associated inhibitory mechanisms in a direct manner but may also require alternative mitogenic signals to take over in UM metastasis.

Metastatic and UM cell lines provide a unique model to identify the mechanisms that regulate ERK1/2 activation in UM. Earlier work already showed that ERK1/2 phosphorylation in UM depends on the MAPK pathway, although mutations in the usual suspects (e.g., *BRAF* and *NRAS*) are lacking (Calipel *et al*, 2006). We investigated the possibility of a tyrosine kinase with differential activities in UM and UM metastasis to be responsible for ERK1/2 activation, using an array of kinase activity assays. Src was revealed as a differentially activated tyrosine kinase and this was supported by incubation with Src-specific kinase inhibitors, PP1 and PP2. Moreover, by treating cell lysates instead of cell cultures, we minimised the secondary effects of the inhibitors. However, PP1 and PP2 affect most of the Src family of tyrosine kinases, and the observed reduction in kinase activity therefore does not specifically mark Src. Multi-target inhibitors are a problem in molecular analysis but may be beneficial in clinical application, as, in CM, a switch from Src to Yes signalling has been reported in brain metastases (Summy and Gallick, 2003). To specifically inhibit Src, we targeted the Src gene expression with a siRNA approach. We detected a reduced kinase activity in Mel270 on transfection and this was correlated with a reduced ERK1 activation. The ERK2 activation seemed unaffected, which could be because of the limited efficacy of siRNA treatment, or it could indicate the activity of another, yet unidentified, kinase. A low Src protein expression in conjunction with a loss of ERK1/2 activation in metastatic UM cell lines, however, supports the hypothesis that, in UM, Src kinase is involved in ERK1/2 activation. Gene expression analysis revealed no significant differences between metastatic and UM cell lines, and thereby indicated that post-transcriptional mechanisms are most likely involved in Src downregulation. Src is both a kinase as well as a client protein for the chaperone HSP90 that is expressed in UM (Missotten *et al*, 2003). Whether HSP90 is reduced in metastases and whether treatment with HSP90 inhibitors depends on Src signalling is part of future investigation (Babchia *et al*, 2008; Faingold *et al*, 2008). The inhibition of Src kinase activity resulted in a strong growth reduction in all UM cultures, whereas in UM cell lines, the response varied more widely. The genetic background of the cell lines might play a role in the observed variation. However, all UM cell lines displayed Src kinase activity and PP1 sensitivity, irrespective of c-kit upregulation (Mel270) or the *BRAF* V600E (OCM1) and *GNAQ* Q209L (Mel202) mutation status (Lefevre *et al*, 2004; Van Raamsdonk *et al*, 2008). Tissue of UM and UM liver metastasis displayed more or less comparable Src levels. The incubation of lysates with Src inhibitors resulted in a comparable reduction of kinase activity in UM and metastasis tissue. The possibility that there exist Src negative clones in liver metastasis can, however, not be ruled out on the basis of these data. Clinical trials targeting Src kinase activity in UM should therefore anticipate this potential risk.

In conclusion, we have identified a differential ERK1/2 activation in UM and metastatic UM cell lines. Using tyrosine kinase activity profiling, we identified Src as a determinant of ERK1/2 activation and showed that Src expression and kinase activity, together with ERK1/2 activation, are reduced in UM metastases cell lines.

## Figures and Tables

**Table 1:** Tyrosine kinase substrates on the kinase array that were differentially phosphorylated between primary UM cell lines and metastatic cell lines

**Substrate**	**UniProt ID**	**Position**	**Log fold change**	**Adj. *P*-value**	**Kinase**
CDK2	P24941	T14/Y15	5.3	0.00005	Lyn
FRK	P42685	Y387	6.2	0.0002	Unknown
SRC8	Q14247	Y499	4.6	0.006	Src
ENOG	P09104	Y43	4.7	0.01	Src
EFS	O43281	Y253	3.1	0.01	Src
PLCG1	P19174	Y771	4.2	0.01	Syk, Sky, GFRs
CD79A	P11912	Y182/Y188	3.1	0.01	Lyn
PAXI	P49023	Y118	4.0	0.02	FAK, Src, Brk
PAXI	P49023	Y31	4.3	0.03	FAK, Src, Brk

Tyrosine kinase substrate specificities are included.
